# Supplementary material for: Common Bean (Phaseolus vulgaris L.) NAC Transcriptional Factor PvNAC52 Enhances Transgenic Arabidopsis Resistance to Salt, Alkali, Osmotic, and ABA Stress by Upregulating Stress-Responsive Genes
Source: Int J Mol Sci. 2024 May 27;25(11):5818. doi: 10.3390/ijms25115818 (PMC11172058; doi:10.3390/ijms25115818)
Supplement: Supplementary file 1 [file ijms-25-05818-s001.zip › ijms-3020804-supplementary.pdf]

Table S1: Primers used in this study.

Genes name	Primer sequence
Phvul.005G084500-F	GCAGGGAACACACCCAATAC
Phvul.005G084500-R	GCACCCAGGTTCTCAATGTT
PvACT11-F	TGCATACGTTGGTGATGAGG
PvACT11-R	AGCCTTGGGGTTAAGAGGAG
PvIDE-F	GCAACCAACCTTTCATCAGC
PvIDE-R	AGAAATGCCTCAACCCTTTG
ATTUB2-F	AGCCTTACAACGCTACTCTGT
ATTUB2-R	CAAATCACCAAAGCTGGGGG
ATACT11-F	ACTCCTGCCATGTATGTCGC
ATACT11-R	TGCAAGGTCCAAACGCAGAA
PvNAC52-F	ATTTCATTTGGAGAGAACACGGGGGACTCTAGAGGATCCC CGGGATGGGAGTTCCAGAG
PvNAC52-R	TTGCTCACCATAAGGGACTGACCACCCGGGGAATGCCTGA ACCC GAATCCCACCGGCTG
SOS1-F	GATTTGTCCCCACGAATGAGA
SOS1-R	TGCGAAGAAGGCGTAGAACA
P5CS1-F	CAAGATGAGATTACATTCG
P5CS1-R	GGTTATGATGACAGGAAT
RD29A-F	GAGCTGTTCAAAGTGAAGTAC
RD29A-R	GTTATACACATGACCACGATCAA
NCED3-F	CAGCACTCAATTCAAGCACTTG
NCED3-R	GTTGTAGGGCTGGTTTGGG
ABI3-F	TGTGACGACTCTTCTGGTGC
ABI3-R	TCATCGGAACAACGACCTGG
ABI5-F	CCAGCTCCGCTTTGTAGGAA
ABI5-R	CCTCCATTACCGCTACCACC
AtDREB1A-F	TCAATTTCGCTGACTCGGCT
AtDREB1A-R	TCCGCCGTGTAAATAGCCTC
AtDREB2A-F	AAGTACCTGCGAAAGGGTCG
AtDREB2A-R	CCAAAGCCTGCTACCTCGAT
AtLEA3-F	GAACGCCGTTTTCAGACGAG
AtLEA3-R	CTTGAAGCTTCCCCAGCTCT
AtLEA7-F	CAAAGACAAGACAGGAAGC
AtLEA7-R	CTCTGTGTCTCACGAGTAG
AtLEA14-F	TTCGATTCCGATCTGTGAG
AtLEA14-R	CACCAACGTCTCGAGCCAG
SOD-F	GGCCAATCTTTGACCCTTTA
SOD-R	AGTCCAGGAGCAAGTCCAGT
MSD1-F	GAAGAACCTTGCTCCTTCCAG
MSD1-R	GATTGGCAGTTGTGTCAACAAC
FSD1-F	GAGAGCTTCTTGCTTTGCTTG
FSD1-R	CATGCTCCCAGACATCAATG

CSD1-F	GATGGTAAAACACACGGTGC
CSD1-R	GCCAGGCTGAGTTCATGGCCTC
POD-F	TTAGGGAGCAGTTTCCCACT
POD-R	AGGGTGAAAGGGAACATCAG
PRX57-F	TCCGAGAAAACTGCTGGACC
PRX57-R	GAGACGGAGATCGTTGGACC
PRX69-F	AAGTCGCGTGGTTCTCCAA
PRX69-R	CCGACGAATCTCCCCATCTG
PRX22-F	AGCGACATGTCCGTGTTCTT
PRX22-R	TGTTCTCACGTGCGCATTTT
CAT-F	TGATCGCGAGAAGATACCTG
CAT-R	CTTCCACGTTCATGGACAAC
ProDH-F	GATCTGCTGCGGTGGGAATA
ProDH-R	TCCTTCCATGAGCTGCTTCG
P5CDH1-F	CGTTGGCCTTATGGTCCTGT
P5CDH1-R	AAGTCCACATCTTCAGCGGG
P5CS2-F	ATCTGTGAACAGCTTGCGGA
P5CS2-R	CAACACCAGCACAAGCCTTC
pGBKT7-PvNAC52-F	GGAATTCATGGGAGTTCCAGA
pGBKT7-PvNAC52-R	CGGGATCCATGCCTGAAC
pGBKT7-PvNAC52-N	CGGGATCCCTTCTTGTATATACGAC
pGBKT7-PvNAC52-C	GGAATTCAACTCGAGTGCACAG
pBGKT7-T7	GTAATACGACTCACTATAGGGCGAGCCGCC
pBGKT7-3'BD	TAAGAGTCACTTTAAAATTGTATAC
PvNAC52-644 bp-Anti-sense	CTGTATTGGGTGTGTTCCCTGCTCG
PvNAC52-475 bp-Anti sense	TGCCTTTCGGAGCTTTGCCAACGT
PvNAC52-614 bp-sense	ACGACGTTCTGGAATCG
PvNAC52-534 bp-sense	GAGGAGAAGGTGAACATTGAG
pAbAi-F	TCTGTGCTCCTTCCTTCGTTCTTCC
pAbAi-R	ATTTAGTGTGTGTATTTGTGTTTGCGTGTC
pAbAi-ABRE-F	agcttACGTGACGTGACGTGc
pAbAi-ABRE-R	tcgagCACGTCACGTCACGTa
pAbAi-MABRE1-F	agcttAAATGAAATGAAATGc
pAbAi-MABRE1-R	tcgagCATTTCATTTCATTTa
pAbAi-MABRE2-F	agcttAAAAGAAAAGAAAAGc
pAbAi-MABRE2-R	tcgagCTTTTCTTTTCTTTTa

Table S2: Gene sequence of *Phvul.005G084500.*

Genes name	sequence
Phvul.005G084500	ATGGGAGTTCCAGAGAAAGACCCTCTTGCCCAGCTGAGTC<br>TACCTCCTGGTTTTCGATTCTACCCCACCGACGAGGAGCTT<br>CTCGTTCAGTATCTCTGCCGCAAGGTCGCCGGCCACCATTT<br>CTCTCTCCCAATCATTGCTGAAATTGATTTGTACAAGTTCG<br>ACCCATGGGTTCTTCCAAGCAAGGCGATTTTCGGGGAGAA<br>AGAGTGGTACTTTTTCAGCCCTCGAGACAGGAAGTACCCA<br>AACGGGTCTCGACCCAACAGAGTAGCCGGGTCGGGTTATT<br>GGAAAGCCACCGGAACCGACAAGATCATCACCACCGAAG<br>GTAGAAAAGTGGGCATAAAAAAAGCCCTGGTTTTTTACGT<br>TGGCAAAGCTCCGAAAGGCACCAAAACCAATTGGATCAT<br>GCACGAGTATCGGCTTCTTGATTCTTCCCGAAAGACCACTG<br>GTACCAAGCTGGATGATTGGGTTCTGTGTCGTATATACAAG<br>AAGAACTCGAGTGCACAGAAGGCGGTGCAAAACGGCGTC<br>GTTTCGAGCAGGGAACACACCCAATACAGCAACGGTTCCT<br>CGTCGTCGTCATCGTCCCATCTGGACGACGTTCTGGAATCG<br>CTGCCAACGATCGACGAACGGTGTTTCATGATGCCACGTG<br>CTCACACGGTGCAGCAACAGCATGAGGAGAAGGTGAACA<br>TTGAGAACCTGGGTGCGGGTGGGTTGGTGGATTGGGCGAA<br>CCCTGCGGTTCTGAATCCGGTGGGTGATTTCGTTTCGGGGA<br>ATAATCAAGTGGTGCAGGAGCATACTCAGGGGATGGTGA<br>ACTACAGCGGGTGCAATGACCTTTATGTTCCAACCTTCTGC<br>CACGTGGAGTCTGCGCTTCCGCAAAAGATGGAGGAAGAG<br>GTGCAAAGCGGCGTGAGAAACCAAAACAGTAATAACTCG<br>TGGTTTCTTCAGAACGATTTTACGCAGGGGTTTCAGAACTC<br>GGTGGACACGTGTGGGTTTAAGTTCCCGGTTCAGCCGGTG<br>GGATTCGGGTTCAGGCATTGA

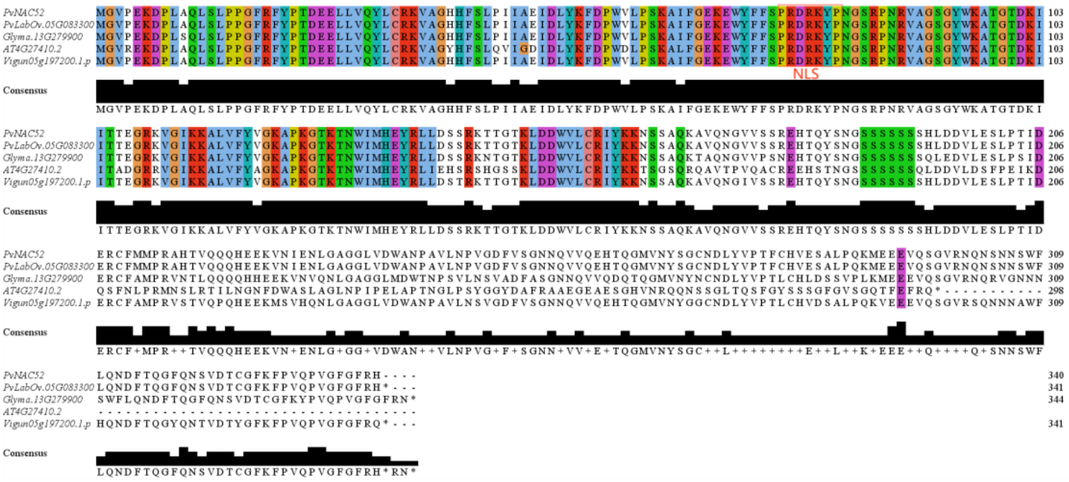

Figure S1. *PvNAC52* gene sequence analysis. All NAC (NAM, ATAF1/2, and CUC1/2) protein sequences used for sequence analysis were retrieved from the Phytozome 13 (https://phytozome-next.jgi.doe.gov/).

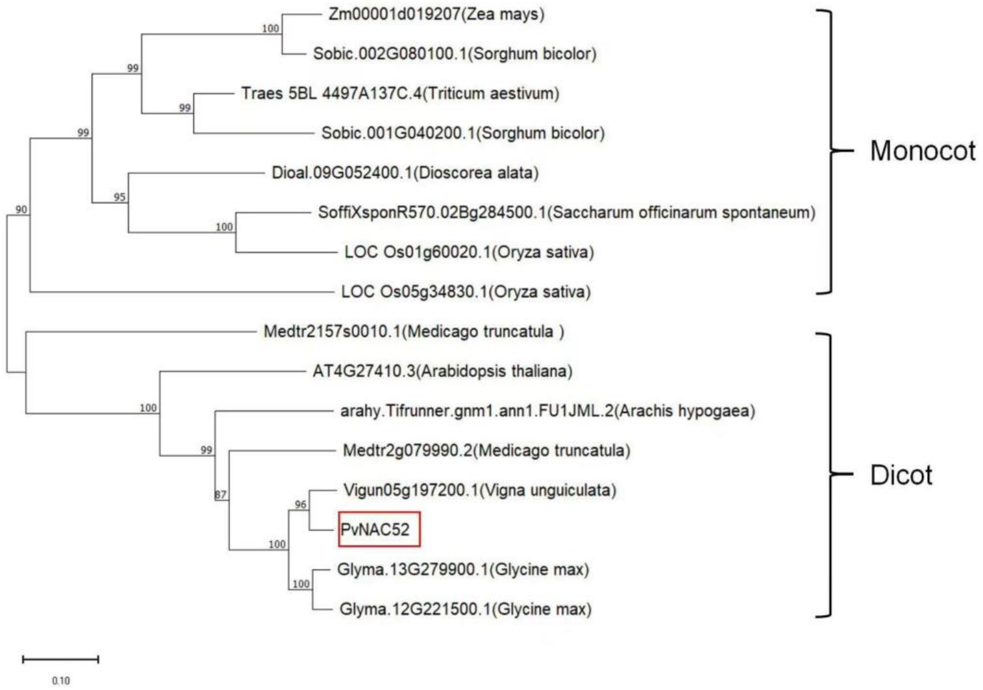

Figure S2. Phylogenetic tree analysis of PvNAC52. All NAC (NAM, ATAF1/2, and CUC1/2) protein sequences used for the phylogenetic analysis were retrieved from the Phytozome 13 (https://phytozome-next.jgi.doe.gov/).

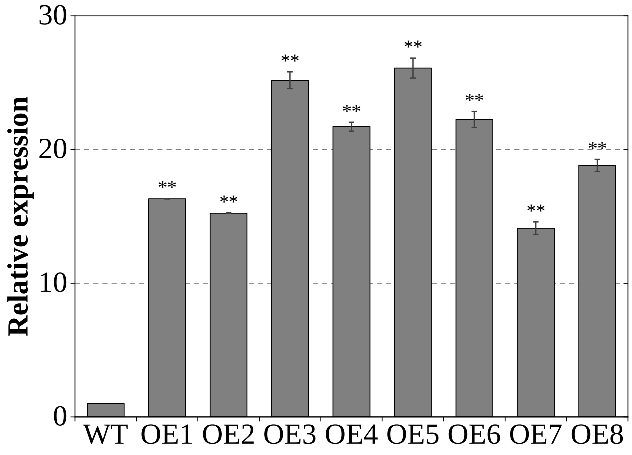

Figure S3. Expression analysis of *PvNAC52* in transgenic lines. Asterisks represent significant differences in relative expression compared to those in WT plant (**P<0.01).

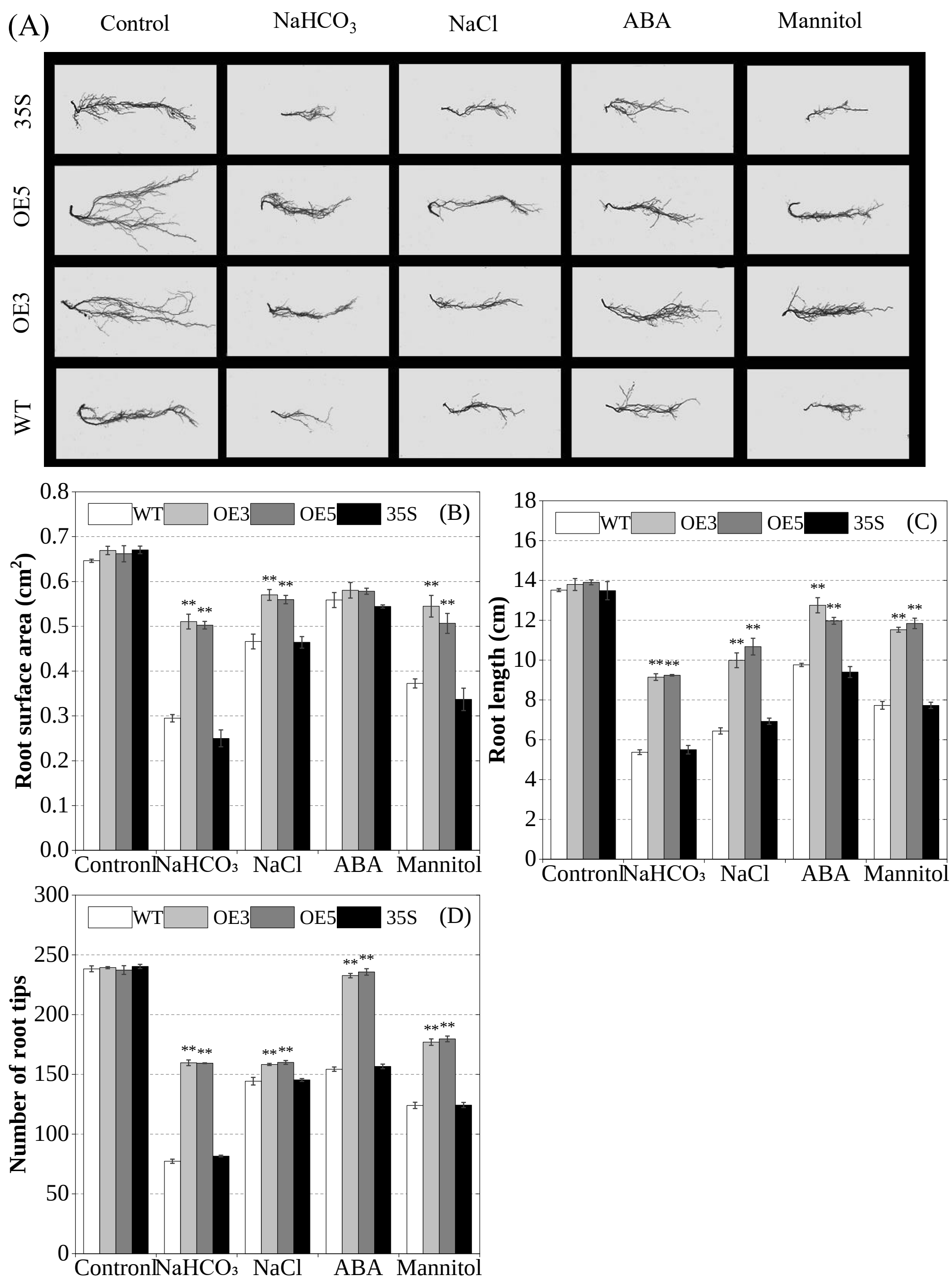

Figure S4. Analysis of root growth under different treatments. (A) Scanning image of four plant roots; (B) root surface area; (C) root length; and (D) number of root tips. Untreated seedlings were used as controls; asterisks represent significant differences compared with the WT plants(**P<.01).

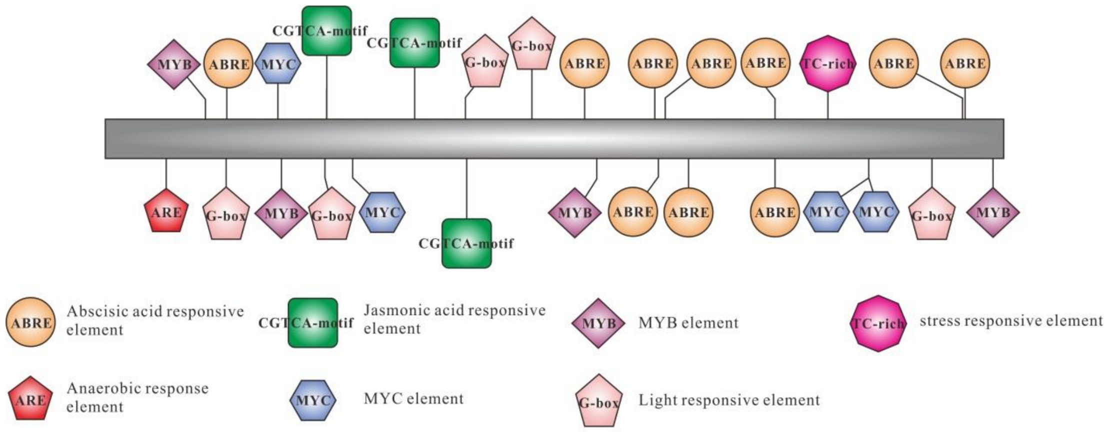

Figure S5. Prediction of *PvNAC52* promoter cis-acting elements.
